# Draft Genome Sequence of *Mycobacterium arupense* Strain GUC1

**DOI:** 10.1128/genomeA.00630-15

**Published:** 2015-06-11

**Authors:** Alexander L. Greninger, Gail Cunningham, Joanna M. Yu, Elaine D. Hsu, Charles Y. Chiu, Steve Miller

**Affiliations:** Department of Laboratory Medicine, UCSF, San Francisco, California, USA

## Abstract

We report the draft genome sequence of *Mycobacterium arupense* strain GUC1 from a sputum sample of a patient with bronchiectasis. This is the first draft genome sequence of *Mycobacterium arupense*, a rapidly growing nonchromogenic mycobacteria.

## GENOME ANNOUNCEMENT

*Mycobacterium arupense* is a rapidly growing nonchromogenic mycobacteria that is closely related to the *Mycobacterium terrae* complex and has been isolated from clinical samples, most commonly sputum samples, as well as environmental water sources (1–3). Multiple reports of tenosynovitis and osteoarticular infections with *M. arupense* have also been presented, including infections caused by the type strain AR30097 (4–8). Although the unique identification of *M. arupense* has generally been related to sequence analysis, the phenotypic properties of *M. arupense* that resulted in it being classified as a species include its inability to grow at 42°C, rapid growth at 30°C, variable pyrazinamidase activity, and mycolic acid patterns that distinguish it from *M. terrae* (1).

Rapidly growing mycobacteria constitute a commonly isolated population of acid-fast bacillus in the clinical microbiology lab of varying clinical importance (9, 10). We sequenced the first draft genome of *M. arupense* from a sputum sample of a patient diagnosed with bronchiectasis. The isolate was originally typed as *M. terrae* complex by high-performance liquid chromatography; however, genome sequencing and analysis of the 16S and *rpoB* sequences revealed its identity as *M. arupense*.

DNA from *M. arupense* strain GUC1 was extracted using the Qiagen EZ1 kit, and paired-end libraries were prepared using the Nextera XT DNA library kit followed by sequencing on the Illumina MiSeq. Sequences were adapter and quality (Q20) trimmed using cutadapt, *de novo* assembled using SPAdes v3.5, metagenomically screened for contaminating sequence with SURPI, and annotated via prokka v1.1 (11–14). A total of 6,386,174 paired-end reads of average length 117 nucleotides were recovered after trimming. *De novo* assembly yielded 173 contigs for a total assembly size of 4,441,412 bp with an *N*_50_ of 56,189 bp, an average coverage of 115×, and a total of 4,182 coding sequences. Contiguity was most likely disrupted by the high G+C content (67%) along with several high-copy-number integrases, transposases, and recombinases that were longer than sequence read length. Other high-copy number contigs included those containing genes to ESX/type VII secretion system, a distantly related 3-methyladenine glycosylase, and a copper-transporting ATPase. The assembly also includes 44 kb across two contigs that aligns with 99 to 100% nucleotide identity to the pMK12478 plasmid from *Mycobacterium kansasii* strain ATCC 12478 (15). Otherwise, the closest aligning sequenced genomes were *Mycobacterium* sp. JDM601 or *Mycobacterium avium* strains E1/E93 at approximately 80% nucleotide identity. By Comprehensive Antibiotic Resistance Database analysis, the GUC1 strain includes an *ampC* beta-lactamase and two metallo-beta-lactamases which demonstrate 80%, 90%, and 77% amino acid identity to that of *M. avium* strain Env 77, respectively (16, 17).

### Nucleotide sequence accession numbers.

This whole-genome shotgun project has been deposited at DDBJ/ENA/GenBank under the accession no. LASW00000000. The assembly described in this paper is the second version, LASW02000000.

## References

[1] Cloud JL, Meyer JJ, Pounder JI, Jost KC, Sweeney A, Carroll KC, Woods GL 2006 *Mycobacterium arupense* sp. nov., a non-chromogenic bacterium isolated from clinical specimens. Int J Syst Evol Microbiol 56:1413–1418. doi:10.1099/ijs.0.64194-0. 16738122

[2] Liu R, Yu Z, Zhang H, Yang M, Shi B, Liu X 2012 Diversity of bacteria and mycobacteria in biofilms of two urban drinking water distribution systems. Can J Microbiol 58:261–270. doi:10.1139/w11-129. 22356469

[3] Castillo-Rodal AI, Mazari-Hiriart M, Lloret-Sánchez LT, Sachman-Ruiz B, Vinuesa P, López-Vidal Y 2012 Potentially pathogenic nontuberculous mycobacteria found in aquatic systems. Analysis from a reclaimed water and water distribution system in Mexico City. Eur J Clin Microbiol Infect Dis 31:683–694. doi:10.1007/s10096-011-1359-y. 21805195

[4] Senda H, Muro H, Terada S 2011 Flexor tenosynovitis caused by *Mycobacterium arupense*. J Hand Surg Eur Vol 36:72–73. doi:10.1177/1753193410381825. 21169302

[5] Seidl A, Lindeque B 2014 Large joint osteoarticular infection caused by *Mycobacterium arupense*. Orthopedics 37:e848–e850. doi:10.3928/01477447-20140825-93. 25350631

[6] Beam E, Vasoo S, Simner PJ, Rizzo M, Mason EL, Walker RC, Deml SM, Brown-Elliott BA, Wallace RJ, Wengenack NL, Sia IG 2014 *Mycobacterium arupense* flexor tenosynovitis: case report and review of antimicrobial susceptibility profiles for 40 clinical isolates. J Clin Microbiol 52:2706–2708. doi:10.1128/JCM.00277-14. 24789193 PMC4097750

[7] Tsai T, Lai C, Tsai I, Chang C, Hsiao C, Hsueh P 2008 Tenosynovitis caused by *Mycobacterium arupense* in a patient with diabetes mellitus. Clin Infect Dis 47:861–863. doi:10.1086/591281. 18713040

[8] Legout L, Ettahar N, Massongo M, Veziris N, Ajana F, Beltrand E, Senneville E 2012 Osteomyelitis of the wrist caused by *Mycobacterium arupense* in an immunocompetent patient: a unique case. Int J Infect Dis 16:e761–e762. doi:10.1016/j.ijid.2012.05.007. 22809581

[9] Greninger AL, Langelier C, Cunningham G, Keh C, Melgar M, Chiu CY, Miller S 29 4 2015 Two rapidly growing mycobacterial species isolated from a brain abscess: first whole genome sequence of *Mycobacterium immunogenum* and *Mycobacterium llatzerense*. J Clin Microbiol. doi:10.1128/JCM.00402-15. PMC4473206 25926490

[10] De Groote MA, Huitt G 2006 Infections due to rapidly growing mycobacteria. Clin Infect Dis 42:1756–1763. doi:10.1086/504381. 16705584

[11] Seemann T 2014 Prokka: rapid prokaryotic genome annotation. Bioinformatics 30:2068–2069. doi:10.1093/bioinformatics/btu153. 24642063

[12] Naccache SN, Federman S, Veeraraghavan N, Zaharia M, Lee D, Samayoa E, Bouquet J, Greninger AL, Luk K-C, Enge B, Wadford DA, Messenger SL, Genrich GL, Pellegrino K, Grard G, Leroy E, Schneider BS, Fair JN, Martínez MA, Isa P, Crump JA, DeRisi JL, Sittler T, Hackett J, Miller S, Chiu CY 2014 A cloud-compatible bioinformatics pipeline for ultrarapid pathogen identification from next-generation sequencing of clinical samples. Genome Res 24:1180–1192. doi:10.1101/gr.171934.113. 24899342 PMC4079973

[13] Martin M 2011 Cutadapt removes adapter sequences from high-throughput sequencing reads. EMBnet.journal 17:10–12. doi:10.14806/ej.17.1.200.

[14] Bankevich A, Nurk S, Antipov D, Gurevich AA, Dvorkin M, Kulikov AS, Lesin VM, Nikolenko SI, Pham S, Prjibelski AD, Pyshkin AV, Sirotkin AV, Vyahhi N, Tesler G, Alekseyev MA, Pevzner PA 2012 SPAdes: a new genome assembly algorithm and its applications to single-cell sequencing. J Comput Biol 19:455–477. doi:10.1089/cmb.2012.0021. 22506599 PMC3342519

[15] Wang J, McIntosh F, Radomski N, Dewar K, Simeone R, Enninga J, Brosch R, Rocha EP, Veyrier FJ, Behr MA 2015 Insights on the emergence of *Mycobacterium tuberculosis* from the analysis of *Mycobacterium kansasii*. Genome Biol Evol 7:856–870. doi:10.1093/gbe/evv035. 25716827 PMC5322544

[16] Hsu C, Wu C, Talaat AM 2011 Genome-wide sequence variation among *Mycobacterium avium* subspecies *paratuberculosis* isolates: a better understanding of Johne’s disease transmission dynamics. Front Microbiol 2:236. doi:10.3389/fmicb.2011.00236. 22163226 PMC3234532

[17] McArthur AG, Waglechner N, Nizam F, Yan A, Azad MA, Baylay AJ, Bhullar K, Canova MJ, De Pascale G, Ejim L, Kalan L, King AM, Koteva K, Morar M, Mulvey MR, O’Brien JS, Pawlowski AC, Piddock LJV, Spanogiannopoulos P, Sutherland AD, Tang I, Taylor PL, Thaker M, Wang W, Yan M, Yu T, Wright GD 2013 The comprehensive antibiotic resistance database. Antimicrob Agents Chemother 57:3348–3357. doi:10.1128/AAC.00419-13. 23650175 PMC3697360

